# B-vitamin Supplementation Mitigates Effects of Fine Particles on Cardiac Autonomic Dysfunction and Inflammation: A Pilot Human Intervention Trial

**DOI:** 10.1038/srep45322

**Published:** 2017-04-03

**Authors:** Jia Zhong, Letizia Trevisi, Bruce Urch, Xinyi Lin, Mary Speck, Brent A. Coull, Gary Liss, Aaron Thompson, Shaowei Wu, Ander Wilson, Petros Koutrakis, Frances Silverman, Diane R. Gold, Andrea A. Baccarelli

**Affiliations:** 1 Columbia University Mailman School of Public Health, New York, New York, USA; 2 Department of Environmental Health, Harvard T.H. Chan School of Public Health, Boston, Massachusetts, USA; 3 Division of Occupational & Environmental Health, Dalla Lana School of Public Health, University of Toronto, Toronto, Ontario, Canada; 4 Singapore Institute for Clinical Sciences, Singapore; 5 Department of Biostatistics, Harvard T.H. Chan School of Public Health, Boston, Massachusetts, USA; 6 Department of Occupational and Environmental Health Sciences, School of Public Health, Peking University, Beijing, 100191 China; 7 Department of Medicine, University of Toronto, Toronto, Ontario, Canada; 8 Li Ka Shing Knowledge Institute, St Michael’s Hospital, Toronto, Ontario, Canada; 9 Southern Ontario Centre for Atmospheric Aerosol Research, Toronto, Ontario, Canada; 10 Channing Laboratory, Brigham and Women’s Hospital, Harvard Medical School, Boston, Massachusetts, USA

## Abstract

Ambient fine particle (PM_2.5_) pollution triggers acute cardiovascular events. Individual-level preventions are proposed to complement regulation in reducing the global burden of PM_2.5_–induced cardiovascular diseases. We determine whether B vitamin supplementation mitigates PM_2.5_ effects on cardiac autonomic dysfunction and inflammation in a single-blind placebo-controlled crossover pilot trial. Ten healthy adults received two-hour controlled-exposure-experiment to sham under placebo, PM_2.5_ (250 μg/m^3^) under placebo, and PM_2.5_ (250 μg/m^3^) under B-vitamin supplementation (2.5 mg/d folic acid, 50 mg/d vitamin B_6_, and 1 mg/d vitamin B_12_), respectively. At pre-, post-, 24 h-post-exposure, we measured resting heart rate (HR) and heart rate variability (HRV) with electrocardiogram, and white blood cell (WBC) counts with hematology analyzer. Compared to sham, PM_2.5_ exposure increased HR (3.8 bpm, 95% CI: 0.3, 7.4; *P* = 0.04), total WBC count (11.5%, 95% CI: 0.3%, 24.0%; *P* = 0.04), lymphocyte count (12.9%, 95% CI: 4.4%, 22.1%; *P* = 0.005), and reduced low-frequency power (57.5%, 95% CI: 2.5%, 81.5%; *P* = 0.04). B-vitamin supplementation attenuated PM_2.5_ effect on HR by 150% (*P* = 0.003), low-frequency power by 90% (*P* = 0.01), total WBC count by 139% (*P* = 0.006), and lymphocyte count by 106% (*P* = 0.02). In healthy adults, two-hour PM_2.5_ exposure substantially increases HR, reduces HRV, and increases WBC. These effects are reduced by B vitamin supplementation.

Ambient fine particulate matter (PM_2.5_) pollution contributes to 3.7 million premature deaths per year worldwide, predominantly through acute effects on the cardiovascular system^1^. Indeed, PM pollution is the most frequent trigger for myocardial infarction at the population level^2^. Even at levels below the current National Ambient Air Quality Standards (NAAQS), associations of PM_2.5_ exposure with increased cardiovascular risk have been found in sensitive individuals^3^,^4^. Moreover, many urban areas outside of North America continue to have elevated levels of PM_2.5_ pollution^1^,^5^. Reducing the global burden of cardiovascular disease (CVD) due to PM_2.5_ pollution requires defined options for individual-level prevention that complement regulatory measures^4^.

Reduced heart rate variability (HRV), reflecting a perturbation in autonomic function^6^,^7^, is a sensitive marker that changes rapidly in response to PM_2.5_ exposure^3^. It represents a primary pathophysiologic intermediate that may proceed PM-related adverse cardiovascular events^4^. In the Normative Aging Study, we found associations of reduced HRV with short-term PM_2.5_ exposure that were limited to subjects with lower intakes of vitamin B_6_ or B_12_ and were abrogated in those with higher intakes^6^. These findings suggest that B vitamins provide protection against the effect of PM_2.5_ on the autonomic nervous system.

Previous epidemiologic studies have implicated B vitamin levels (folic acid, vitamins B_6_ and B_12_) in CVD susceptibility^8^,^9^. However, to date, the results from randomized clinical trials do not support the benefit of B vitamin supplementation for CVD prevention^10^,^11^,^12^,^13^,^14^,^15^. Recent studies suggest that B vitamins may minimize health effects of environmental stressors through their anti-inflammatory and antioxidant properties^6^,^16^. In animal models, B vitamin supplementation has been successfully used to curb oxidative stress, inflammation, and metabolic phenotype change due to environmental stressors^17^,^18^. However, no clinical trial has yet investigated whether B vitamin supplementation alters the biologic response to ambient air pollution exposure.

To the best of our knowledge, we established the first trial to evaluate whether B vitamin supplementation can attenuate the acute autonomic effects of PM_2.5_ using a single-blind crossover intervention with controlled exposure to fine concentrated ambient particles (fine CAP, i.e., PM_2.5_) in ten healthy adults. We investigated the PM_2.5_ effect on HRV and, because of the central role of inflammation in modulating the cardiovascular effects of PM_2.5_, on total and differential white blood cell (WBC) counts, as well as the potential for B vitamins to counteract these effects.

## Results

### Study population and exposure levels

**Figure 1:**
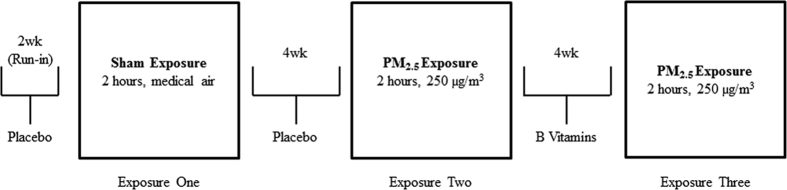
Study design: A single-blind, cross-over intervention trial with controlled exposure experiments in ten healthy volunteers^19^.

As previously described^19^, all volunteers (baseline characteristics described in Supplementary Table 1) completed three controlled exposure experiments (July 2013 to February 2014) (Fig. 1). The baseline resting HR ranged from 43.0 to 74.0 bmp (median, 58.9 bpm), and did not vary substantially by age, gender, race, or being overweight.

The target PM_2.5_ concentrations were controlled by design; however, there was some variations in the actual PM_2.5_ concentration (Supplementary Table 2). Among all controlled exposures to PM_2.5_, the concentration varied from 100.6 to 287.5 μg/m^3^ (median, 234.0 μg/m^3^). Previous studies using the same exposure facility reported minimal PM_2.5_ concentration in medical air (median, 0.0 μg/m^3^; interquartile range, 2.40 μg/m^3^)^20^. No significant difference in PM_2.5_ concentration existed between exposure 2 and 3 (*P* = 0.38). During the study period, the 7-day moving average of ambient PM_2.5_ level was 9.30 ± 0.36 μg/m^3^ in the study area.

### Plasma concentrations of B vitamins

Four-week B vitamin supplementation significantly increased plasma concentrations of folic acid, vitamins B_6_ and B_12_ (*P* = 0.02, *P* = 0.004, *P* = 0.01; respectively), while placebo had no effect (*P* = 0.82, *P* = 0.75, *P* = 0.42, respectively) (Supplementary Table 3).

### Effect of PM_2.5_ on heart rate (HR), HRV, and WBC without B vitamin supplement

**Figure 2:**
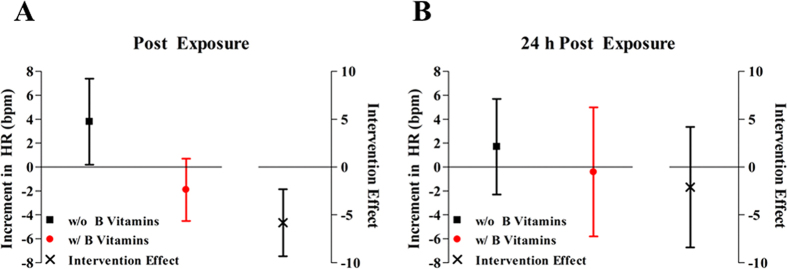
Increment in heart rate (HR) associated with PM_2.5_, and the intervention effect of B vitamin supplementation. The intervention effect represents the difference in estimated PM_2.5_ effects between exposure 2 and exposure 3 (due to B vitamin supplementation). Results were adjusted for chamber humidity, chamber temperature, and season (Spring/Summer/Fall/Winter).

**Figure 3:**
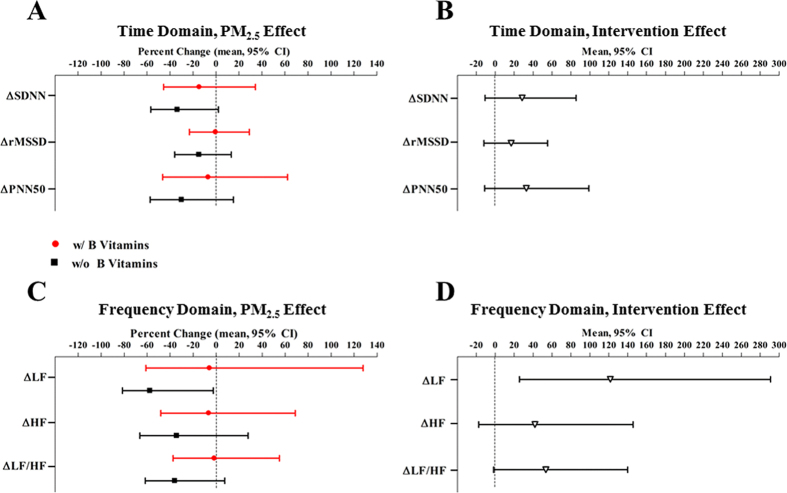
Immediate change of heart rate variability (HRV) associated with PM_2.5_, and the intervention effect of B vitamin supplementation. SDNN indicates the standard deviation of normal-to-normal (NN) intervals; rMSSD indicates the root mean square of successive differences; PNN50 indicates percentage of differences between adjacent NN intervals that are greater than 50 milliseconds; LF indicates low-frequency power (0.04–0.15 Hz); HF indicates high-frequency power (0.15–0.4 Hz). Panel A and C represents the % change in post-exposure HRV/pre-exposure HRV ratio associated with PM_2.5_ exposure, compared to medical air. Results were adjusted for chamber humidity, chamber temperature, and season (Spring/Summer/Fall/Winter).

**Figure 4:**
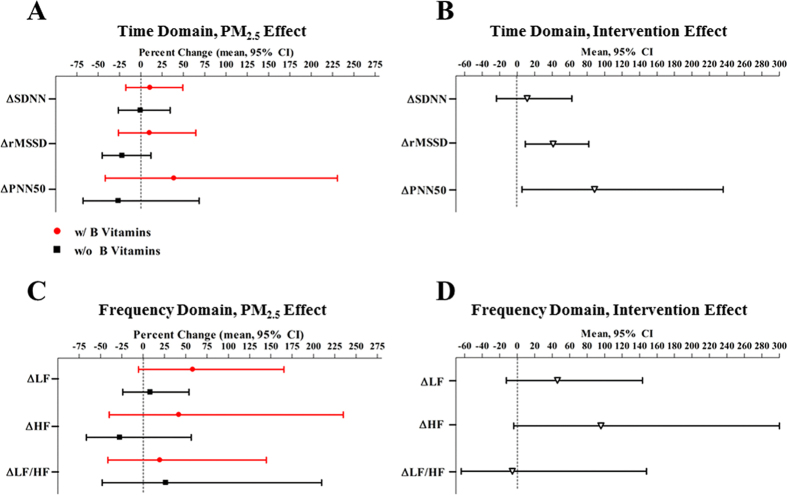
Twenty-four h post-exposure change of heart rate variability (HRV) associated with PM_2.5_, and the intervention effect of B vitamin supplementation. SDNN indicates the standard deviation of normal-to-normal (NN) intervals; rMSSD indicates the root mean square of successive differences; PNN50 indicates percentage of differences between adjacent NN intervals that are greater than 50 milliseconds; LF indicates low-frequency power (0.04–0.15 Hz); HF indicates high-frequency power (0.15–0.4 Hz). Panel A and C represents the % change in 24 h post-exposure HRV/pre-exposure HRV ratio associated with PM_2.5_ exposure, compared to medical air. Results were adjusted for chamber humidity, chamber temperature, and season (Spring/Summer/Fall/Winter).

In the absence of B vitamin supplement, HR increased (Fig. 2) and HRV decreased (Fig. 3) after PM_2.5_ exposure. Compared to sham, two-hour PM_2.5_ exposure was associated with 3.8 bpm (95% CI, 0.3 bpm, 7.4 bpm; *P* = 0.04) higher resting HR. PM_2.5_ exposure was associated with 33.6% (95% CI, −2.1%, 56.8%; *P* = 0.06), 57.5% (95% CI, 2.5%, 81.5%; *P* = 0.04), and 35.9% (95% CI, −7.5%, 61.8%; *P* = 0.09) lower standard deviation of NN intervals (SDNN), low-frequency (LF) power, and low-frequency/high-frequency (LF/HF) ratio compared to sham, respectively (Supplementary Table 4). At 24 h post-exposure, we did not observe any significant effect of PM_2.5_ exposure on HR or HRV (Fig. 4 and Supplementary Table 5).

**Figure 5:**
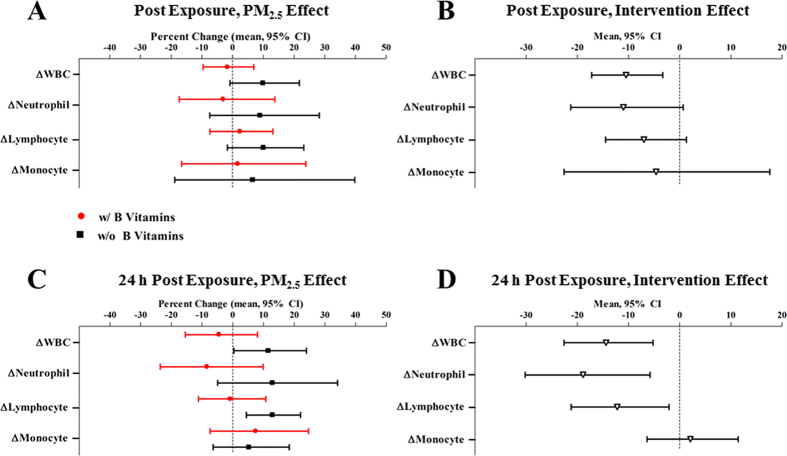
Change of total and differential white blood cell (WBC) counts associated with PM_2.5_, and the intervention effect of B vitamin supplementation. Panel A and C represents the % change in post-exposure cell count/pre-exposure cell count ratio associated with PM_2.5_ exposure, compared to medical air. Results were adjusted for chamber humidity, chamber temperature, and season (Spring/Summer/Fall/Winter).

Exposure to PM_2.5_ increased total and differential WBC counts, compared to sham (Fig. 5). Immediately after exposure, PM_2.5_ was non-significantly associated with 9.9% (95% CI, −0.8%, 21.8%; *P* = 0.07) and 10.0% (95% CI, −1.7%, 23.2%; *P* = 0.09) higher total WBCs and lymphocytes. Twenty-four hours later, PM_2.5_ exposure was significantly associated with 11.5% (95% CI, 0.3%, 24.0%; *P* = 0.04) and 12.9% (95% CI, 4.4%, 22.1%; *P* = 0.005) higher total WBCs, neutrophils, lymphocytes, and monocytes, respectively (Supplementary Table 6).

### B vitamin supplementation attenuated the effects of PM_2.5_

After four-week B vitamin supplementation, the associations of PM_2.5_ with outcomes, for example, post-exposure HR (*P*_intervention_ = 0.003), HRV (*P*_intervention_ = 0.01 for LF), and total WBC count (*P*_intervention_ = 0.008), were significantly attenuated. The effect of PM_2.5_ on HR was no longer significant with B vitamin supplementation (−1.9 bpm, 95% CI, −4.6 bpm, 0.7 bpm; *P* = 0.14) (Fig. 2). Likewise, B vitamin supplementation reduced the effect size of PM_2.5_ by 90% for LF and 96% for LF/HF ratio (Fig. 3). Further, exposure to two-hour PM_2.5_ was associated with 5.8% (95% CI, −127.8%, 61.0%; *P* = 0.89; *P*_intervention_ = 0.01) and 1.6% (95% CI, −55.0%, 37.5%; *P* = 0.94; *P*_intervention_ = 0.06) lower LF and LF/HF ratio, respectively, with B vitamin supplementation (Supplementary Table 4). In addition, although non-significant, B vitamin supplementation attenuated the PM_2.5_ effect by 57% on SDNN, 97% on square root of the mean squared differences of successive NN intervals (rMSSD), 77% on proportion of successive NN intervals with differences >50 msec (pNN50), and 81% on HF (Fig. 3).

The attenuation of the PM_2.5_-HR or PM_2.5_-HRV relationship (*P*_intervention_ = 0.003, 0.01, 0.03 for HR, rMSSD, and PNN50, respectively) by B vitamins remained significant at 24 h post-exposure (Fig. 4).

The associations of PM_2.5_ with post-exposure total and differential WBC counts were also weakened by B vitamin supplementation (Fig. 5). Compared to sham, effects of PM_2.5_ on WBCs were non-significant in the presence of B vitamin supplementation: two-hour PM_2.5_ exposure was associated with a −1.7% (95% CI, −9.6%, 6.9%; *P* = 0.67; *P*_intervention_ = 0.008), −3.1% (95% CI, −17.4%, 13.8%; *P* = 0.68; *P*_intervention_ = 0.06), and 2.4% (95% CI, −7.4%, 13.1%; *P* = 0.62; *P*_intervention_ = 0.09) change in total WBCs, neutrophils, and lymphocytes, respectively (Supplementary Table 6). In summary, B vitamin supplementation reduced the PM_2.5_ effect by 117%, 134%, 76%, and 75% on total WBCs, neutrophils, lymphocytes, and monocytes, respectively (Fig. 5).

Likewise, B vitamin supplementation attenuated the PM_2.5_ effects on total and differential WBC counts at 24 h post-exposure (*P*_intervention_ = 0.006, 0.01, 0.02, 0.61 for total WBC, neutrophil, lymphocyte, and monocyte) (Supplementary Table 6). B-vitamin supplementation significantly reduced the PM_2.5_ effect by 139% on total WBC, 165% on neutrophil, and 106% on lymphocyte (Fig. 5).

### Sensitivity analysis

We dealt with potential influence by season by adjusting for spring/summer/fall/winter in all models. In a sensitivity analysis, we additionally adjusted for seasonality (defined using sine and cosine functions)^21^ to further address residual confounding, and our conclusions remained the same (data not shown). We observed no significant changes in dietary intake of folic acid, vitamins B_6_, and B_12_ during the study period, therefore confounding due to dietary B vitamins was minimized (Supplementary Table 7). To rule out the possibility that the observed change in HRV was partially due to HR fluctuation, we adjusted for HR in PM_2.5_-HRV analysis and obtained similar results (data not shown). In addition, we conducted sensitivity analysis using HR-normalized HRV measurements and our conclusions were consistent (Supplementary Table 8).

## Discussion

This single-blind crossover intervention trial with controlled exposure experiments found that two-hour exposure to concentrated ambient PM_2.5_ (250 μg/m^3^) has substantial physiologic impacts on HR, HRV, and WBCs among healthy adults. Further, we demonstrated that these effects are nearly abolished with four-week B-vitamin supplementation.

With ambient PM_2.5_ levels far exceeding NAAQS in many urban megacities worldwide^22^, pollution regulation remains the backbone of public health protection against its cardiovascular health effects. Indeed, improved cardiovascular health, reflected in reduced morbidity and mortality, has been documented as pollution levels have decreased in the U.S^23^. Nevertheless, even in U.S. cities complying with NAAQS, cardiovascular effects of particle pollution have been observed, with no evidence for a threshold for effect in sensitive individuals^3^,^4^,^6^,^22^. Thus, the medical and public health communities have sought adjunct personal measures that might complement regulation in reducing the cardiovascular risk of pollution in sensitive people^24^.

Previous studies suggested that dietary supplementations with vitamins C, vitamins E, or polyunsaturated fatty acids might protect against short-term air pollution-induced adverse cardiopulmonary responses^25^,^26^,^27^. In a randomized double-blinded controlled exposure study, Tong and coauthors successfully demonstrated that a four-week fish oil supplementation attenuated CAP-induced HRV reductions^26^. Our choice to assess the potential protective benefits of B vitamin supplementation against PM-induced cardiac autonomic dysfunction and inflammation was motivated by the anti-inflammatory, antioxidant, and immunoepigenetic effects of B vitamins^28^,^29^. Recent epidemiological and *in vivo* studies suggest that B vitamins might be particularly protective against air pollution-induced cardiovascular effects—as it was demonstrated to modulate the epigenetic and inflammatory signaling pathways linking air pollution, intermediate biomarkers, and cardiovascular outcomes^6^,^17^,^27^,^28^. For example, folic acid and vitamin B_6_ lower chemokine release from peripheral blood mononuclear cells and circulating levels of pro-inflammatory molecules^28^,^29^, indicating reduced risk for acute cardiovascular events such as stroke. In mice model, folic acid protects against lipopolysaccharide-induced nuclear factor-kβ pathway activation and adverse birth outcomes^16^. Furthermore, B vitamins are essential nutrients involved in the biochemical process of DNA methylation^3^. In the presence of air pollution, adequate B vitamin intake ensures proper epigenetic status of leukocytes to warrant proper immuno-regulation, and prevents excessive oxidative damage to the cardiovascular system^3^. Although the results of randomized controlled trials on supplementation with folic acid, vitamin B_6_ and B_12_ do not support benefits of B vitamins for either primary or secondary CVD prevention^12^,^14^,^15^,^30^, the mentioned interactive biological properties of B vitamins render it a promising preventive strategy to minimize the cardiovascular damage due to ambient PM_2.5_ pollution. However, no prior clinical investigation has tested whether B vitamin supplementation can be used to guard the cardiovascular system from the adverse health effects of PM_2.5_.

Our findings of a primary autonomic effect of PM_2.5_ are consistent with previous human controlled exposures studies^31^,^32^,^33^,^34^, showing that short-term PM_2.5_ exposure perturbed cardiorespiratory autonomic function as reflected in increased HR and reduced HRV^6^. Immediately following two-hour exposure to ambient concentrated PM_2.5_, we observed a substantial increase in resting HR and a reduction in LF power. These results indicate a consistent reduction in HRV across five measures – which reflects the adverse pathophysiological modulations in cardiac autonomic control by PM_2.5_ exposure.

PM_2.5_ is a potent trigger for leukocyte-mediated inflammation, which is proposed as the key mechanism underlying the pathological modulation of the cardiovascular system by PM_2.5_ exposure^4^. Our data support this hypothesis by showing that two-hour PM_2.5_ exposure triggers increased total WBC count and lymphocyte count at 24-hour post exposure. In healthy adults, PM pollution increases the number of neutrophils and lymphocytes in alveolar lavage and peripheral blood^35^. While the underlying biological mechanism remains unclear, *in vivo* studies suggest that PM stimulates bone marrow via alveolar macrophages-mediated cytokine signaling, leading to accelerated release of immature leukocytes in to the circulation^36^,^37^.

Twenty-four hours after exposure, the effect of PM_2.5_ on HR and HRV weakened. However, PM_2.5_ exposure remained significantly associated with higher numbers of total WBCs and lymphocytes. Taken together, although the acute physiological responses due to PM_2.5_ exposure peak might be reversible, the pro-inflammatory effects of PM_2.5_ appears to be sustained beyond 24 hours and represent a biomarker that could have clinical relevance to sensitive individuals in a community setting^4^.

For the first time, our trial provides evidences demonstrating the unique preventive benefits of B vitamin administration in the context of air pollution: B vitamin supplementation can diminish the acute effects of PM_2.5_ on cardiac autonomic dysfunction and inflammatory markers. These findings are in agreement with our results from the Normative Aging Study^6^ – a population with average B vitamins intakes well above the standard dietary references – in which short-term PM_2.5_ exposure was associated with lower HRV (7.1% reduction in SDNN per 10 μg/m^3^ increase in PM_2.5_), and the adverse effect of PM_2.5_ was limited to subjects with lower (<median) intakes of vitamin B_6_, vitamin B_12_, or methionine.

This study has several strengths, including its crossover design with controlled exposure experiments – which simulate conditions similar to urban air pollution peaks, while allowing for control of exposure and treatment at the individual level. The Harvard ambient particle concentrators do not affect the concentration of gaseous pollutants, therefore, minimizing the confounding due to gaseous co-pollutants such as ozone and sulfur dioxide. All exposure experiments were conducted at the same time of day to eliminate impacts of diurnal variation. We adjusted for time-varying factors including season, chamber temperature and humidity to minimize their influence on the observed associations, while time invariant factors are controlled by the crossover design.

We acknowledge several limitations, however. We determined the number to recruit in the current study using power estimates that are penalized by conservative Bonferroni’s adjustments for multiple HRV indexes. Although our sample size is comparable to previous controlled CAP exposure studies, which succeeded in demonstrating health effects of CAP exposure^32^,^33^,^38^,^39^, our study is evidently limited in power to detect small effects with only 10 subjects (30 controlled exposure experiments).

Further, treatment sequence could not be randomized due to the long half-life of B vitamins, therefore might be subject to confounding by period or ordering effects. For example, the first exposure experiment is likely to produce more distress on volunteers because of psychological effect. We intentionally provided medical air as the first exposure experiment; therefore the psychological effect is expected to bias the effect of PM_2.5_ towards the null. The short study duration with four-week intervals between exposure experiments also reduced the impact of temporal trends. In addition, we contrasted the post- *vs* pre-exposure status to ascertain all outcome measurements, which is expected to be less prone to confounding due to temporal trend than the absolute values. While residual confounding is possible, considering the magnitude of our effect estimates and the consistency across different HRV index, it is unlikely that the observed association reflected bias from confounding. The crossover design of the present study was not complete, as we had no arm of B vitamin supplementation with sham exposure. Therefore, separating the direct effect of B vitamins on cardiac autonomic dysfunction and inflammation (i.e., in the absence of air pollution) from the combined effect of both B vitamins and air pollution can be statistically challenging and requires strong assumptions. In addition, our study was limited to healthy adults from lightly polluted urban environment, therefore our findings might not be generalizable to populations that are at higher risk for pollution-induced cardiovascular effects (eg, children, older adults, individuals with pre-existing cardiovascular disease, and individuals residing in heavily polluted areas).

Apart from avoiding exercising outdoors at peak pollution times, sensitive individuals have limited options to reduce exposure and associated cardiovascular risk. While regulation is the backbone of prevention, residual risk remains for those who are sensitive, and high exposures are, unfortunately, the rule still in many megacities throughout the world. The present study provides novel experimental evidence showing that an ambient PM_2.5_ exposure peak has unfavorable effect on cardiac autonomic function and the immune system, which can be counteracted by B vitamin supplementation. Our project inaugurates a line of research for the development of preventive pharmacological interventions using B vitamins to contain the health effects of air pollution. Future studies will identify the precise pathophysiological processes of PM-induced cardiovascular responses and inflammation, as well as the mechanistic pathway underlying the protective effect of B vitamins.

## Methods

### Study population and sample size

We recruited ten healthy, 18–60-year-old, non-smoking volunteers who were not on any form of B vitamin supplementation or other medication, from the University of Toronto St. George campus and surrounding area (downtown Toronto, Ontario, Canada)^19^. The number to recruit was determined through power calculation based on a 2-sided alternative at α = 0.05/6 = 0.0833 to reflect a Bonferroni correction for multiple testing (six HRV indexes). We estimate 80% (90%) power to detect correlations with absolute magnitude of 0.40 (0.45) in ten volunteers with two repeated measures, which is less than or equal to the magnitude of the correlations reported in our previous studies^33^,^38^,^39^. The trial and experimental protocols were approved by all participating institutional review board (University of Toronto, St. Michael’s Hospital, and Harvard T.H. Chan School of Public Health) and registered (clinicaltrials.gov NCT01864824, date of registration: May 8, 2013). All methods were performed in accordance with the relevant guidelines and regulations. All volunteers provided written informed consent. The conduct of the trial was monitored by an independent data and safety monitoring committee.

### Study design

We conducted a single-blind placebo-controlled crossover pilot trial (Fig. 1)^19^ with controlled exposure experiments (July 2013 to February 2014). A two-hour sham exposure experiment (exposure one, particle-free medical air) was included to provide baseline data. All volunteers then received placebo for four weeks preceding the two-hour exposure experiment to concentrated ambient PM_2.5_ (exposure two, 250 μg/m^3^). After exposure two, we administered B vitamin supplements for four weeks before the next two-hour exposure experiment to PM_2.5_ (exposure three, 250 μg/m^3^). The four-week placebo or B vitamin treatment also served as washout periods between exposure experiments to diminish the carryover effect of PM_2.5_ exposure^19^,^40^, while minimizing the impact of seasonality and temporal trend on the source and composition of the concentrated ambient PM_2.5_. To ensure comparable conditions across all controlled exposure experiments to PM_2.5_, the present study could not randomize on the treatment (placebo *vs* B vitamins) sequence because vitamin B_12_ has a biological half-life longer than four months^41^. Study volunteers were blinded to exposure and treatment allocation. Based on our symptom survey, none of the volunteers was able to discern the exposure type for any exposure experiment.

### Exposure facility

Harvard fine particle concentrators with a dilution control system delivered target-concentration PM_2.5_^42^, and the sham exposures with medical air were generated as previously described^38^. The concentrated ambient PM_2.5_ air stream was delivered directly to the volunteer who was seated inside a 4.9 m^3^ (1.1 × 1.9 × 2.0 m) Lexan enclosure, at rest and breathing normally via an “oxygen type” facemask covering his/her nose and mouth. During each exposure experiment to PM_2.5_, particles were collected on Teflon filters for monitoring gravimetric determination of PM_2.5_ exposure mass concentration (μg/m^3^).

### Folic acid, vitamin B_6_ and B_12_ supplement

During three four-week periods, we administered one B vitamin tablet (2.5 mg folic acid, 50 mg vitamin B_6_, and 1 mg vitamin B_12_) or placebo daily. The placebo tablets contain identical non-medical ingredients as the B vitamin tablets. Tablet preparation and packaging were done by an external lab (Jamieson Laboratory, Toronto, Canada). The label coding was blinded to the volunteers. We monitored volunteers’ plasma folic acid and vitamin B_6_ and B_12_ levels before each exposure experiment. A self-administered validated semi-quantitative Food Frequency Questionnaire was used to assess dietary B vitamin intake at the first and last visits to rule out potential impact from diet.

### Heat rate, heart rate variability, and WBC measurement

We measured supine resting HR and HRV as the primary outcome before (pre-exposure) and after (immediately post-exposure and 24 h post-exposure) each exposure experiment, using high-resolution (1 KHz sample rate) digital 12-lead Holter electrocardiogram monitors (H12 + recorder, Mortara Instruments, Milwaukee, WI). We extracted ten-minute HRV readings on time domain outcomes (SDNN, rMSSD, pNN50), and frequency domain outcomes (LF power, HF power, and LF/HF ratio). We discarded the first three minutes and the last two minutes during the ten-minute recording and analyzed the remaining five-minute electrocardiogram data using standardized techniques^43^. SDNN represents the total variability. PNN50, rMSSD, and HF are sensitive to high-frequency heart rate fluctuations and are considered as measures of cardiac vagal tone modulation, while LF power is linked to the activity of both sympathetic and parasympathetic nervous system.

Blood samples (pre-, post-, and 24 h post-exposure) were obtained in ethylenediaminetetraacetic acid vacutainer tubes, stored at 4 °C, and subsequently processed in a local laboratory within two hours for total and differential WBC counts using the Technicon H-1 automated hematology analyzer (Technicon Instruments Corp, Tarrytown, NY, USA).

### Statistical methods

We conducted graphical explorations and log_10_-transformed the HRV measures and WBC counts to improve normality and stabilize the variance. We examined the linear relationships between HR/HRV/WBC and all independent variables and covariates, and observed no deviation from linearity. For the ease of interpretation, we scaled the effect estimates to the percent changes in HRV and WBC in all models.

We used linear mixed-effects models with a robust/sandwich estimator for the variance (Model 1) to account for within-subject correlation in the outcome measures. Random intercepts were assigned to each subject. In all models, we adjusted for covariates with potential influences on HR, HRV, and WBC – selected based on prior knowledge and the existing literature – season (fall/winter/spring/summer), chamber temperature, and relative humidity.


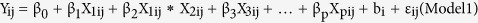


In the above model, *Y*_*ij*_ was the change in HR, HRV, or WBC (i.e., ΔHR = post-exposure HR – pre-exposure HR) for participant *i* at exposure occasion *j. β*_*0*_ was the overall intercept, and *b*_*i*_ was the separate random intercept for subject *i* with, *b*_*i*_ ~ N(0, ϴ), *ε*_*ij*_ ~ N(0, σ^2^). *X*_*1ij*_ was a binary variable indicating exposure to PM_2.5_ or medical air. *X*_*2ij*_ was a binary variable indicating placebo or B vitamin supplementation. *X*_*3ij*_*–X*_*pij*_ were the covariates, for participant *i* at measurement *j*. The main effect of B vitamin supplementation was not included in the model, given volunteers did not receive any medical air exposure while on B vitamin supplementation. *β*_*1*_ represents the effect of PM_2.5_ exposure without B vitamin supplementation and *β*_*1 +*_*β*_*2*_ represents the effect of PM_2.5_ exposure with B vitamin supplementation. *β*_*2*_ thus represents the intervention effect of B vitamin supplementation (i.e, the attenuation of PM_2.5_ effect due to B vitamin supplementation). A two tailed value of *P* ≤ 0.05 was considered statistically significant. We represent the *P* value for the intervention effect, *β*_*2,*_by *P*_intervention_. Analyses were performed using SAS 9.4 (SAS Institute, Cary NC).

## Additional Information

**How to cite this article:** Zhong, J. *et al*. B-vitamin Supplementation Mitigates Effects of Fine Particles on Cardiac Autonomic Dysfunction and Inflammation: A Pilot Human Intervention Trial. *Sci. Rep.* **7**, 45322; doi: 10.1038/srep45322 (2017).

**Publisher's note:** Springer Nature remains neutral with regard to jurisdictional claims in published maps and institutional affiliations.

## Supplementary Material

Supplementary Materials
