# Supplementary material for: B-vitamin Supplementation Mitigates Effects of Fine Particles on Cardiac Autonomic Dysfunction and Inflammation: A Pilot Human Intervention Trial
Source: Sci Rep. 2017 Apr 3;7:45322. doi: 10.1038/srep45322 (PMC5377246; doi:10.1038/srep45322)
Supplement: Supplementary Materials [file srep45322-s1.pdf]

B-vitamin Supplementation Mitigates Effects of Fine Particles on Cardiac Autonomic Dysfunction and Inflammation: A Pilot Human Intervention Trial

Jia Zhong[1*], Letizia Trevisi[2], Bruce Urch[3], Xinyi Lin[4], Mary Speck[3], Brent A. Coull[5], Gary Liss[3], Aaron Thompson[3], Shaowei Wu[6], Ander Wilson[5], Petros Koutrakis[2], Frances Silverman[3,7-9], Diane R. Gold[2,10], and Andrea A. Baccarelli[1]

Zhong; Air Pollution, B Vitamins, and Trial

[1]Columbia University Mailman School of Public Health, New York, New York, USA
[2]Department of Environmental Health, Harvard T.H. Chan School of Public Health, Boston, Massachusetts, USA
[3]Division of Occupational & Environmental Health, Dalla Lana School of Public Health, University of Toronto, Toronto, Ontario, Canada;
[4]Singapore Institute for Clinical Sciences, Singapore
[5]Department of Biostatistics, Harvard T.H. Chan School of Public Health, Boston, Massachusetts, USA;
[6]Department of Occupational and Environmental Health Sciences, School of Public Health, Peking University, Beijing, China 100191
[7]Department of Medicine, University of Toronto, Toronto, Ontario, Canada
[8]Li Ka Shing Knowledge Institute, St Michael's Hospital, Toronto, Ontario, Canada
[9]Southern Ontario Centre for Atmospheric Aerosol Research, Toronto, Ontario, Canada
[10]Channing Laboratory, Brigham and Women's Hospital, Harvard Medical School, Boston, Massachusetts, USA.
*Corresponding Author
Jia Zhong, ScD
Department of Environmental Health Sciences
Columbia University Mailman School of Public Health
630 West 168th Street, New York, NY 10032
E-mail: jiazhong@mail.harvard.edu
Phone: (202) 305-3067
Fax: (617) 432-6913

Supplementary Table 1 Baseline characteristics of the study participants (N=10)

Characteristics	N (%)	Resting Heart Rate Mean (SD)
Age (years)		
19-29	7 (70)	62.3 (8.1)
30-49	3 (30)	51.0 (7.0)
Gender		
Female	6 (60)	61.0 (8.9)
Male	4 (40)	55.8 (10.0)
Race		
White	4 (40)	52.0 (6.2)
Asian	3 (30)	64.3 (8.5)
Other	3 (30)	62.7 (9.5)
BMI (kg/m^2)		
<25	7 (70)	62.1 (8.2)
≥25	3 (30)	51.3 (7.6)

SD, standard deviation; BMI, body mass index.

Supplementary Table 2 Total exposure mass concentration of fine particles ($PM_{2.5}$) across three exposure experiments

Exposure*	Mean (SD)	Median (IQR)	Minimum	Maximum	*P*†
Exposure 1 (Medical Air + Placebo) ($\mu g/m^3$)	--	--	--	--	--
Exposure 2 ($PM_{2.5}$ + Placebo) ($\mu g/m^3$)	217.1 (50.6)	219.1 (33.1)	100.6	287.5	
Exposure 3 ($PM_{2.5}$ + B vitamins) ($\mu g/m^3$)	233.8 (31.2)	237.2 (48.7)	184.6	286.2	0.38

SD, standard deviation; IQR, inter-quartile range.
*Integrated gravimetric (filter sample) exposure concentrations sampled from $PM_{2.5}$ airstream inlet to human exposure chamber.
†*P* value from the paired T test.

Supplementary Table 3 Plasma concentration of folate, vitamin B_6, and vitamin B_{12}, before each exposure experiment

	Mean (SD)	Median (IQR)	Minimum	Maximum	P^*
	Before Exposure 1 (Medical Air + Placebo)				
Folate (nmoles/L)	37 (9)	35 (14)	28	56	--
B_6 (nmoles/L)	42 (11)	41 (16)	25	57	--
B_{12} (pmoles/L)	305 (107)	292 (72)	173	486	--
	Before Exposure 2 ($PM_{2.5}$ + Placebo)				
Folate (nmoles/L)	39 (13)	39 (24)	22	56	0.82
B_6 (nmoles/L)	87 (142)	37 (18)	19	482	0.75
B_{12} (pmoles/L)	302 (99)	262 (214)	197	441	0.42
	Before Exposure 3 ($PM_{2.5}$ + B vitamins)				
Folate (nmoles/L)	124 (186)	56 (13)	27	638	0.02
B_6 (nmoles/L)	393 (224)	428 (321)	23	708	0.004
B_{12} (pmoles/L)	507 (166)	511 (85)	176	794	0.01

SD, standard deviation; IQR, inter-quartile range.
*P value from the Wilcoxon signed-rank test; the measurement taken before exposure 1 was used as the reference group.

Supplementary Table 4 Immediate change of post- *vs* pre-exposure heart rate (HR) and heart rate variability (HRV) associated with two-hour exposure to fine particles ($PM_{2.5}$), and the attenuated $PM_{2.5}$ effect due to B vitamin supplementation (N=10)

	Effect of $PM_{2.5}$ without B vitamin supplementation			Effect of $PM_{2.5}$ with B vitamin supplementation			Intervention Effect		
	Estimate	*P*	95% CI	Estimate	*P*	95% CI	Estimate	$P_{intervention}$ †	95% CI
ΔHR (bpm)	3.8	0.04	0.3 to 7.4	-1.9	0.14	-4.6 to 0.7	-5.8	0.003	-9.2 to -2.3
	% Change*	*P*	95% CI	% Change*	*P*	95% CI	% Change*	$P_{intervention}$ †	95% CI
Time Domain									
ΔSDNN	-33.6	0.06	-56.8 to 2.1	-14.6	0.47	-45.6 to 34.3	28.6	0.16	-10.8 to 85.4
ΔrMSSD	-14.8	0.25	-35.9 to 13.3	-0.4	0.97	-23.1 to 28.9	16.9	0.26	-12.0 to 55.3
ΔPNN50	-29.9	0.15	-57.4 to 15.2	-6.8	0.79	-46.4 to 62.2	33.0	0.15	-11.1 to 98.9
Frequency Domain									
ΔLF	-57.5	0.04	-81.5 to -2.5	-5.8	0.89	-61.0 to 127.8	121.6	0.01	25.7 to 290.8
ΔHF	-34.3	0.2	-66.2 to 27.7	-6.5	0.81	-48.2 to 68.8	42.0	0.19	-17.5 to 145.8
ΔLF/HF	-35.9	0.09	-61.8 to 7.5	-1.6	0.94	-37.5 to 55.0	53.6	0.06	-1.6 to 140.0

HR indicates heart rate; SDNN indicates the standard deviation of normal-to-normal (NN) intervals; rMSSD indicates the root mean square of successive differences; PNN50 indicates percentage of differences between adjacent NN intervals that are greater than 50 milliseconds; LF indicates low-frequency power (0.04 –0.15 Hz); HF indicates high-frequency power (0.15–0.4 Hz).

*Represents the % change in post-exposure HRV/pre-exposure HRV ratio associated with $PM_{2.5}$ exposure, compared to medical air. Results were adjusted for chamber humidity, chamber temperature, and season (Spring/Summer/Fall/Winter).

†Represents the intervention effect of B vitamin supplementation (or effect modification by B vitamin supplementation).

Supplementary Table 5 Change of 24-hour post- *vs* pre-exposure heart rate (HR) and heart rate variability (HRV) associated with two-hour exposure to fine particles ($PM_{2.5}$), and the attenuated $PM_{2.5}$ effect due to B vitamin supplementation (N=10)

	Effect of $PM_{2.5}$ without B vitamin supplementation			Effect of $PM_{2.5}$ with B vitamin supplementation			Intervention Effect		
	Estimate	*P*	95% CI	Estimate	*P*	95% CI	Estimate	$P_{intervention}$†	95% CI
ΔHR (bpm)	1.7	0.37	-2.3 to 5.7	-0.4	0.89	-5.7 to 5.0	-2.1	0.48	-8.3 to 4.2
	% Change*	*P*	95% CI	% Change*	*P*	95% CI	% Change*	$P_{intervention}$†	95% CI
Time Domain									
ΔSDNN	-0.4	0.98	-26.3 to 34.5	11.0	0.46	-17.6 to 49.4	11.4	0.54	-23.6 to 62.6
ΔrMSSD	-21.9	0.16	-45.4 to 11.8	10.2	0.60	-26.2 to 64.6	41.1	0.01	9.4 to 82.0
ΔPNN50	-26.3	0.43	-67.7 to 68.4	38.9	0.42	-41.7 to 230.9	88.4	0.03	5.7 to 235.7
Frequency Domain									
ΔLF	8.4	0.62	-23.7 to 53.9	58.3	0.08	-5.5 to 165.4	46.1	0.13	-12.3 to 143.4
ΔHF	-27.5	0.38	-66.5 to 56.5	42.0	0.39	-39.8 to 234.9	95.9	0.06	-4.0 to 300.0
ΔLF/HF	26.7	0.57	-48.2 to 209.6	19.8	0.59	-41.3 to 144.5	-5.5	0.90	-63.9 to 147.8

HR indicates heart rate; SDNN indicates the standard deviation of normal-to-normal (NN) intervals; rMSSD indicates the root mean square of successive differences; PNN(10/20/50) indicates percentage of differences between adjacent NN intervals that are greater than (10/20/50) milliseconds; LF indicates low-frequency power (0.04 –0.15 Hz); HF indicates high-frequency power (0.15–0.4 Hz).

*Represents the % change in 24-hour post-exposure HRV/pre-exposure HRV ratio associated with $PM_{2.5}$ exposure, compared to medical air. Results were adjusted for chamber humidity, chamber temperature, and season (Spring/Summer/Fall/Winter).

†Represents the intervention effect of B vitamin supplementation.

Supplementary Table 6 Change of white blood cell (WBC) counts and distribution associated with two-hour exposure to fine particles ($PM_{2.5}$), and the attenuated $PM_{2.5}$ effect due to B vitamin supplementation (N=10)

	Effect of $PM_{2.5}$ without B vitamin supplementation			Effect of $PM_{2.5}$ with B vitamin supplementation			Intervention Effect		
	% Change*	*P*	95% CI	% Change*	*P*	95% CI	% Change*	$P_{intervention}$†	95% CI
				Immediate post-pre change in WBC					
ΔWBC	9.9	0.07	-0.8 to 21.8	-1.7	0.67	-9.6 to 6.9	-10.5	0.008	-17.2 to -3.3
ΔNeutrophil	8.9	0.28	-7.5 to 28.2	-3.1	0.68	-17.4 to 13.8	-11.0	0.06	-21.3 to 0.7
ΔLymphocyte	10.0	0.09	-1.7 to 23.2	2.4	0.62	-7.4 to 13.1	-7.0	0.09	-14.5 to 1.3
ΔMonocyte	6.5	0.62	-18.8 to 39.8	1.6	0.86	-16.6 to 23.9	-4.6	0.63	-22.6 to 17.6
				24-hour post-pre change in WBC					
ΔWBC	11.5	0.04	0.3 to 24.0	-4.5	0.43	-15.5 to 8.0	-14.4	0.006	-22.6 to -5.2
ΔNeutrophil	12.9	0.15	-4.9 to 34.1	-8.4	0.32	-23.6 to 9.9	-18.9	0.01	-30.2 to -5.8
ΔLymphocyte	12.9	0.005	4.4 to 22.1	-0.8	0.87	-11.2 to 10.7	-12.2	0.02	-21.2 to -2.1
ΔMonocyte	5.2	0.36	-6.4 to 18.3	7.5	0.31	-7.3 to 24.7	2.1	0.61	-6.4 to 11.4

*Represents the % change in post-exposure cell count/pre-exposure cell count ratio associated with $PM_{2.5}$ exposure, compared to medical air. Results were adjusted for chamber humidity, chamber temperature, and season (Spring/Summer/Fall/Winter).
†Represents the intervention effect of B vitamin supplementation.

Supplementary Table 7 Dietary intake of folic acid, vitamin B_6, and vitamin B_{12}, at screening and the end of study

	Mean (SD)	Median (IQR)	Range	Mean (SD)	Median (IQR)	Range	*P**
	Screening			End of Study			
Folic acid(mcg/d)	172.8 (105.7)	161.5 (143.0)	17.0 to 376.0	171.8 (121.7)	169.0 (130.0)	3.0 to 429.0	0.98
B_6 (mg/d)	2.1 (0.8)	2.2 (0.6)	0.3 to 3.6	2.0 (0.7)	2.0 (1.1)	0.9 to 3.2	0.86
B_{12} (mcg/d)	6.5 (4.1)	5.6 (4.1)	0.9 to 13.4	5.6 (2.2)	6.8 (2.5)	1.3 to 7.6	0.57

SD indicates standard deviation; IQR indicates inter-quartile range.
**P* value from the paired T test.

Supplementary Table 8 Effect of two-hour exposure to fine particles ($PM_{2.5}$) and intervention effect of B vitamin supplementation on heart rate variability (HRV), normalized to heart rate (HR) (N=10)

	Effect of $PM_{2.5}$ without B vitamin supplement			Effect of $PM_{2.5}$ with B vitamin supplement			Intervention Effect		
	% Change*	*P*	95% CI	% Change*	*P*	95% CI	% Change*	$P_{intervention}$†	95% CI
				Immediate post exposure					
Time Domain									
ΔSDNN	-33.1	0.06	-56.2 to 2.2	-13.4	0.50	-44.8 to 35.9	29.4	0.14	-9.5 to 85.1
ΔrMSSD	-14.4	0.29	-36.9 to 16.3	1.0	0.93	-23.6 to 33.7	18.0	0.27	-13.7 to 61.3
ΔPNN50	-29.5	0.17	-57.9 to 18.0	-5.5	0.84	-46.9 to 68.2	34.0	0.16	-12.1 to 104.6
Frequency Domain									
ΔLF	-57.5	0.04	-81.3 to -3.5	-4.8	0.90	-60.2 to 127.6	124.0	0.007	29.3 to 288.1
ΔHF	-34.0	0.21	-66.7 to 30.8	-5.2	0.85	-48.5 to 74.6	43.7	0.19	-18.7 to 153.8
ΔLF/HF	-35.6	0.07	-60.2 to 4.1	-0.2	0.99	-35.2 to 53.9	55.0	0.03	4.9 to 129.1
				24-hour post exposure					
Time Domain									
ΔSDNN	-3.8	0.79	-29.5 to 31.4	10.9	0.527	-21.7 to 57.0	15.2	0.41	-20.2 to 66.3
ΔrMSSD	-24.3	0.17	-49.9 to 14.5	9.9	0.67	-31.5 to 76.3	45.1	0.04	2.9 to 104.7
ΔPNN50	-28.5	0.42	-70.3 to 72.1	38.7	0.46	-45.8 to 254.9	93.9	0.05	1.1 to 271.9
Frequency Domain									
ΔLF	4.7	0.80	-29.0 to 54.3	58.7	0.09	-8.0 to 173.9	51.6	0.08	-6.5 to 146.0
ΔHF	-29.7	0.36	-68.9 to 58.7	41.5	0.43	-44.2 to 258.8	101.5	0.07	-7.6 to 339.5
ΔLF/HF	22.8	0.61	-48.3 to 191.7	19.4	0.57	-39 to 133.5	-2.8	0.95	-60.1 to 137.0

SDNN indicates the standard deviation of normal-to-normal (NN) intervals; rMSSD indicates the root mean square of successive differences; PNN50 indicates percentage of differences between adjacent NN intervals that are greater than 50 milliseconds; LF indicates low-frequency power (0.04 –0.15 Hz); HF indicates high-frequency power (0.15–0.4 Hz).

[*]Represents the % change in post-exposure HR-normalized HRV/pre-exposure HRV ratio associated with $PM_{2.5}$ exposure, compared to medical air. Results were adjusted for chamber humidity, chamber temperature, and season (Spring/Summer/Fall/Winter).
[†]Represents the intervention effect of B vitamin supplementation (or effect modification by B vitamin supplementation)
